# [^18^F]CB251 PET/MR imaging probe targeting translocator protein (TSPO) independent of its Polymorphism in a Neuroinflammation Model

**DOI:** 10.7150/thno.46875

**Published:** 2020-07-23

**Authors:** Kyungmin Kim, Ha Kim, Sung-Hwan Bae, Seok-Yong Lee, Young-Hwa Kim, Juri Na, Chul-Hee Lee, Min Sun Lee, Guen Bae Ko, Kyeong Yun Kim, Sang-Hee Lee, In Ho Song, Gi Jeong Cheon, Keon Wook Kang, Sang Eun Kim, June-Key Chung, Euishin Edmund Kim, Sun-Ha Paek, Jae Sung Lee, Byung Chul Lee, Hyewon Youn

**Affiliations:** 1 Department of Nuclear Medicine, Seoul National University Hospital, Republic of Korea.; 2 Biomedical Sciences, Cancer Research Institute, Seoul National University College of Medicine, Republic of Korea.; 3 Laboratory of Molecular Imaging and Therapy, Cancer Research Institute, Seoul National University College of Medicine, Republic of Korea.; 4 Department of Nuclear Medicine, Seoul National University Bundang Hospital, Republic of Korea.; 5 Department of Transdisciplinary Studies, Graduate School of Convergence Science and Technology, Seoul National University, Republic of Korea.; 6 Center for Nanomolecular Imaging and Innovative Drug Development, Advanced Institutes of Convergence Technology, Seoul National University, Republic of Korea.; 7 Department of Neurosurgery, Seoul National University Hospital, Republic of Korea.; 8 Brightonix Imaging Inc., National Cancer Institute, Republic of Korea.; 9 Department of Nuclear Medicine, National Cancer Institute, Republic of Korea.

**Keywords:** neuroinflammation, TSPO, polymorphism, PET/MRI, multimodal imaging

## Abstract

The 18 kDa translocator protein (TSPO) has been proposed as a biomarker for the detection of neuroinflammation. Although various PET probes targeting TSPO have been developed, a highly selective probe for detecting TSPO is still needed because single nucleotide polymorphisms in the human TSPO gene greatly affect the binding affinity of TSPO ligands. Here, we describe the visualization of neuroinflammation with a multimodality imaging system using our recently developed TSPO-targeting radionuclide PET probe [^18^F]CB251, which is less affected by TSPO polymorphisms.

**Methods:** To test the selectivity of [^18^F]CB251 for TSPO polymorphisms, 293FT cells expressing polymorphic TSPO were generated by introducing the coding sequences of wild-type (WT) and mutant (Alanine → Threonine at 147^th^ Amino Acid; A147T) forms. Competitive inhibition assay was conducted with [^3^H]PK11195 and various TSPO ligands using membrane proteins isolated from 293FT cells expressing TSPO WT or mutant-A147T, representing high-affinity binder (HAB) or low-affinity binder (LAB), respectively. IC_50_ values of each ligand to [^3^H]PK11195 in HAB or LAB were measured and the ratio of IC_50_ values of each ligand to [^3^H]PK11195 in HAB to LAB was calculated, indicating the sensitivity of TSPO polymorphism. Cellular uptake of [^18^F]CB251 was measured with different TSPO polymorphisms, and phantom studies of [^18^F]CB251-PET using 293FT cells were performed. To test TSPO-specific cellular uptake of [^18^F]CB251, TSPO expression was regulated with pCMV-TSPO (or shTSPO)/eGFP vector. Intracranial lipopolysaccharide (LPS) treatment was used to induce regional inflammation in the mouse brain. Gadolinium (Gd)-DOTA MRI was used to monitor the disruption of the blood-brain barrier (BBB) and infiltration by immune cells. Infiltration of peripheral immune cells across the BBB, which exacerbates neuroinflammation to produce higher levels of neurotoxicity, was also monitored with bioluminescence imaging (BLI). Peripheral immune cells isolated from luciferase-expressing transgenic mice were transferred to syngeneic inflamed mice. Neuroinflammation was monitored with [^18^F]CB251-PET/MR and BLI. To evaluate the effects of anti-inflammatory agents on intracranial inflammation, an inflammatory cytokine inhibitor, 2-cyano-3, 12-dioxooleana-1, 9-dien-28-oic acid methyl ester (CDDO-Me) was administered in intracranial LPS challenged mice.

**Results**: The ratio of IC_50_ values of [^18^F]CB251 in HAB to LAB indicated similar binding affinity to WT and mutant TSPO and was less affected by TSPO polymorphisms. [^18^F]CB251 was specific for TSPO, and its cellular uptake reflected the amount of TSPO. Higher [^18^F]CB251 uptake was also observed in activated immune cells. Simultaneous [^18^F]CB251-PET/MRI showed that [^18^F]CB251 radioactivity was co-registered with the MR signals in the same region of the brain of LPS-injected mice. Luciferase-expressing peripheral immune cells were located at the site of LPS-injected right striatum. Quantitative evaluation of the anti-inflammatory effect of CDDO-Me on neuroinflammation was successfully monitored with TSPO-targeting [^18^F]CB251-PET/MR and BLI.

**Conclusion:** Our results indicate that [^18^F]CB251-PET has great potential for detecting neuroinflammation with higher TSPO selectivity regardless of polymorphisms. Our multimodal imaging system, [^18^F]CB251-PET/MRI, tested for evaluating the efficacy of anti-inflammatory agents in preclinical studies, might be an effective method to assess the severity and therapeutic response of neuroinflammation.

## Introduction

Neuroinflammation is the primary defense mechanism against viral and bacterial infections, toxic metabolites, and injury in the central nervous system (CNS). Several studies have shown that prolonged neuroinflammation is linked to the onset and progression of neurodegenerative conditions such as Alzheimer's and Parkinson's diseases, and multiple sclerosis 1-3. Bidirectional interactions between the CNS and peripheral immune systems adversely affect the CNS 4-6 and are considered the main cause of prolonged neuroinflammation and neurodegenerative diseases 5, 6. These interactions induce not only the activation of microglia and resident immune cells in the brain but also the disruption of the blood-brain barrier (BBB), which results in the migration of peripheral immune cells into the region of CNS neuroinflammation 6-8. Therefore, imaging the region of neuroinflammation and investigating the interactions between CNS immune cells and infiltrating peripheral immune cells are important for understanding neurodegenerative diseases.

Several studies of various imaging techniques have been conducted for identifying the region of neuroinflammation 9-13. Single-photon emission computed tomography (SPECT) using ^99m^Tc-hexamethylpropyleneamineoxime (HMPAO) or ^99m^Tc-ethyl cysteinate dimer (ECD) has been frequently used to visualize the increased blood flow that occurs in neuroinflammation 14, 15. Disruption of the BBB and changes in vascular permeability can also be visualized by ^99m^Tc-DTPA SPECT and gadolinium (Gd)-DTPA magnetic resonance imaging (MRI) 16, 17. Also, [^11^C]arachidonic acid and [^18^F]FDG positron emission tomography (PET) have been used for imaging neuroinflammation reflected by changes in incorporation of arachidonic acid and glucose metabolism, respectively, in neurons and inflammatory cells 18. However, [^18^F]FDG-PET cannot be used for early diagnosis of neuronal diseases because the basal level of [^18^F]FDG uptake is too high in the brain to distinguish neuroinflammation from normal brain regions. Therefore, recent studies have focused on identifying better imaging biomarkers to overcome the limitation of [^18^F] FDG-PET for imaging neuroinflammation.

The translocator protein 18 kDa (TSPO) has been proposed as an attractive target biomarker of neuroinflammation 19-31. TSPO, located in the mitochondrial outer membrane, acts as a peripheral benzodiazepine receptor and a cholesterol transporter. Because activated immune cells have been reported to show increased TSPO expression, TSPO-targeting probes such as [^11^C]PK11195 and [^11^C]PBR28 have been suggested for PET imaging of inflammation. However, these PET probes have limitations, such as nonspecific binding, a low target-to-background ratio, and different binding sensitivities to TSPO polymorphisms 28-31. Although radiochemists have been working on developing improved imaging ligands for targeting TSPO, a highly selectable probe for detecting TSPO is still needed.

Our group recently developed a new imidazopyridine TSPO ligand, [^18^F]CB251, which showed promise as a TSPO radiotracer 26. We introduced fluorine-18 because ^18^F-labelled compounds with a longer half-life (109.8 min for fluorine-18 and 20.3 min for carbon-11) are preferable to ^11^C-labelled compounds for clinical applications. We reported on the synthesis of [^18^F]CB251, which showed a 5.1-times higher TSPO binding affinity than [^11^C]PK11195 26, 31.

In this study, we evaluated the selectivity of the [^18^F]CB251 probe for human TSPO harboring genetic variants, and its ability to reveal activated immune cells in regions of neuroinflammation. We established a mouse model of intracranial lipopolysaccharide (LPS)-induced regional inflammation to visualize neuroinflammation using multimodality imaging. Both CNS and peripheral immune cells infiltrating across the BBB play an important role in neuroinflammation. We isolated peripheral immune cells from spleens of luciferase-expressing transgenic mice and systemically injected them into mice with intracranial LPS-induced regional neuroinflammation, and visualized with bioluminescence imaging (BLI). By comparing TSPO-targeting [^18^F]CB251 PET/MR and peripheral immune cells-targeting BLI, we attempted to determine whether the [^18^F]CB251 probe can quantitatively assess the severity of neuroinflammation and therapeutic response to anti-inflammatory agents.

## Results

### *In vitro* evaluation of [^18^F] CB251 as a probe for targeting TSPO to image activated immune cells

**Figure 1:**
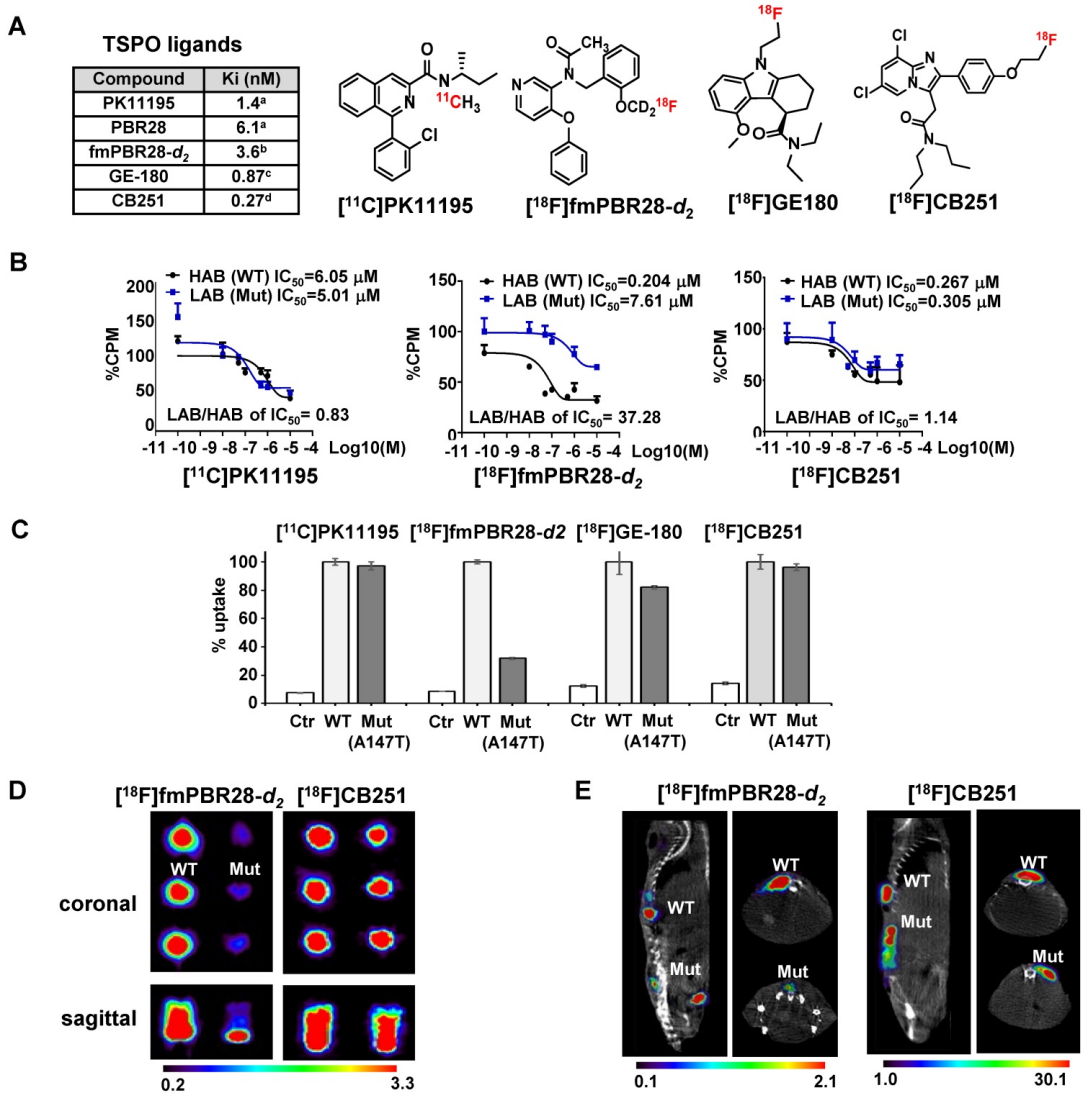
** [^18^F]CB251 as a TSPO-targeting radioactive ligand.** (**A**) Various TSPO ligands and the structure of TSPO-targeting radioactive ligands. CB251 is compared with known TSPO ligands, such as PK11195, PBR28, fm-PBR-d2, and GE-180. [18F]fmPBR28-d2 is fluoromethyl-PBR28-d2 (ref. 24, 30). Ki of ligands for TSPO from refs. 47a, 50b, 31c, and 24d. (**B**) Competitive inhibition assay of PK11195, fmPBR28-d2, and CB251 with [3H]PK11195 on membrane proteins. HAB indicates high affinity ligand binding phenotype (wild type TSPO); LAB indicates low affinity ligand binding phenotype (A147T mutant) isolated from 293FT cells expressing polymorphic TSPOs. CPM indicates count per minute. The ratio of IC50 LAB/HAB represents the sensitivity of TSPO polymorphism; a value close to 1 indicates less sensitive to TSPO polymorphism. (**C**) *In vitro* cellular uptake of CB251 in 293FT cells expressing polymorphic TSPO was compared with TSPO ligands. (**D**) PET scan images of 293FT cells expressing polymorphic TSPOs (**E**) Representative PET/MR images of mice bearing 293FT cells with polymorphic TSPOs (N=2 for each treatment).

As we reported previously 26, an ^18^F-labeled alpidem analog, [^18^F]CB251, was synthesized from 2-phenyl-imidazo [1,2-a] pyridine, and used as a specific probe for TSPO. We also reported that the binding affinity of CB251 was higher than that of the widely used TSPO ligands, PK11195 26, 32, PBR28 26, 32, fluoremethyl-PBR28-*d2* (fmPBR28-*d2*), and GE-180 33 (**Figure 1A**).

For TSPO ligand development, TSPO selectability regardless of TSPO polymorphism (rs6971; A147T) is important because TSPO polymorphism is known to affect both *in vitro* and *in vivo* radio-ligand bindings. TSPO ligands show substantial differences in affinity between subjects classified as a high-affinity binder (HAB; TSPO wild-type (WT)) and low-affinity binder (LAB; TSPO A147T mutant (Mut)). TSPO genotypes and classification is showed in **Figure S1A**. PBR28 was reported to have a different binding affinity to TSPO polymorphism compared to PK11195 28-31.

To test the ligand-binding affinity and cellular uptake, we generated 293FT cells expressing polymorphic TSPOs, including the most abundant type (WT) and mutant TSPO (C to T point mutation generating Alanine to Threonine at 147^th^ amino acid of TSPO; Mut-A147T). **Figure S1B** shows the mutagenesis site and the map of expression vectors.

To compare ligand binding affinity to the TSPO polymorphism, we conducted competitive inhibition assay with [^3^H]PK11195 and various TSPO ligands such as PK11195, fluoromethyl-PBR28-*d_2_* (fmPBR28-*d_2_*) and CB251 using membrane proteins isolated from 293FT cells expressing TSPO polymorphism (TSPO WT and TSPO Mut-A147T) (**Figure 1B**). The ratio of IC_50_ values (IC_50_ of LAB/ IC_50_ of HAB) for PK11195 was 0.83 and similar to earlier reports 34, 35. Unlike fmPBR28-*d_2_* (IC_50_ of LAB/ IC_50_ of HAB=37.28), CB251 showed a similar ratio (IC_50_ of LAB/ IC_50_ of HAB =1.14), which was close to 1. These results indicated that fmPBR28-*d_2_* ligand had a difference in binding affinity according to the TSPO polymorphism, whereas CB251 showed ratios similar to PK11195.

The higher affinity of ligand binding to the target protein is an important factor. However, clinical applications also require consideration of biochemical, cellular, and physiological factors, such as lipophilicity of the ligand and its non-specific insertion in the membrane, cellular metabolism of the ligand, endogenous cellular TSPO levels, and *in vivo* kinetics. Also, the higher affinity of a ligand to the target proteins does not automatically reflect target selectivity. Therefore, we performed cellular uptake of various radiolabeled ligands targeting TSPO because it reflects the entire cellular process from infiltration through the cell membrane to its binding to TSPO on the mitochondrial outer membrane. As expected, our results demonstrated that [^18^F]fmPBR28-*d_2_* was sensitive to the A147T mutation. GE-180, another well-known TSPO ligand, showed less sensitivity to A147T mutation. However, [^11^C]PK1195 and [^18^F]CB251 were much less sensitive to TSPO polymorphism, and [^18^F]CB251 showed similar cellular uptake levels between the polymorphic TSPOs (**Figure 1C**).

We also observed differences in PET signals from the cells with different polymorphic TSPOs using phantoms. [^18^F]fmPBR28-*d_2_*was used as a sensitive probe for TSPO polymorphism. Similar to the cellular uptake test results, [^18^F]CB251 was less sensitive to TSPO polymorphism (**Figure 1D, E**), indicating that it could be a good PET probe with better selectivity for targeting TSPO.

**Figure 2:**
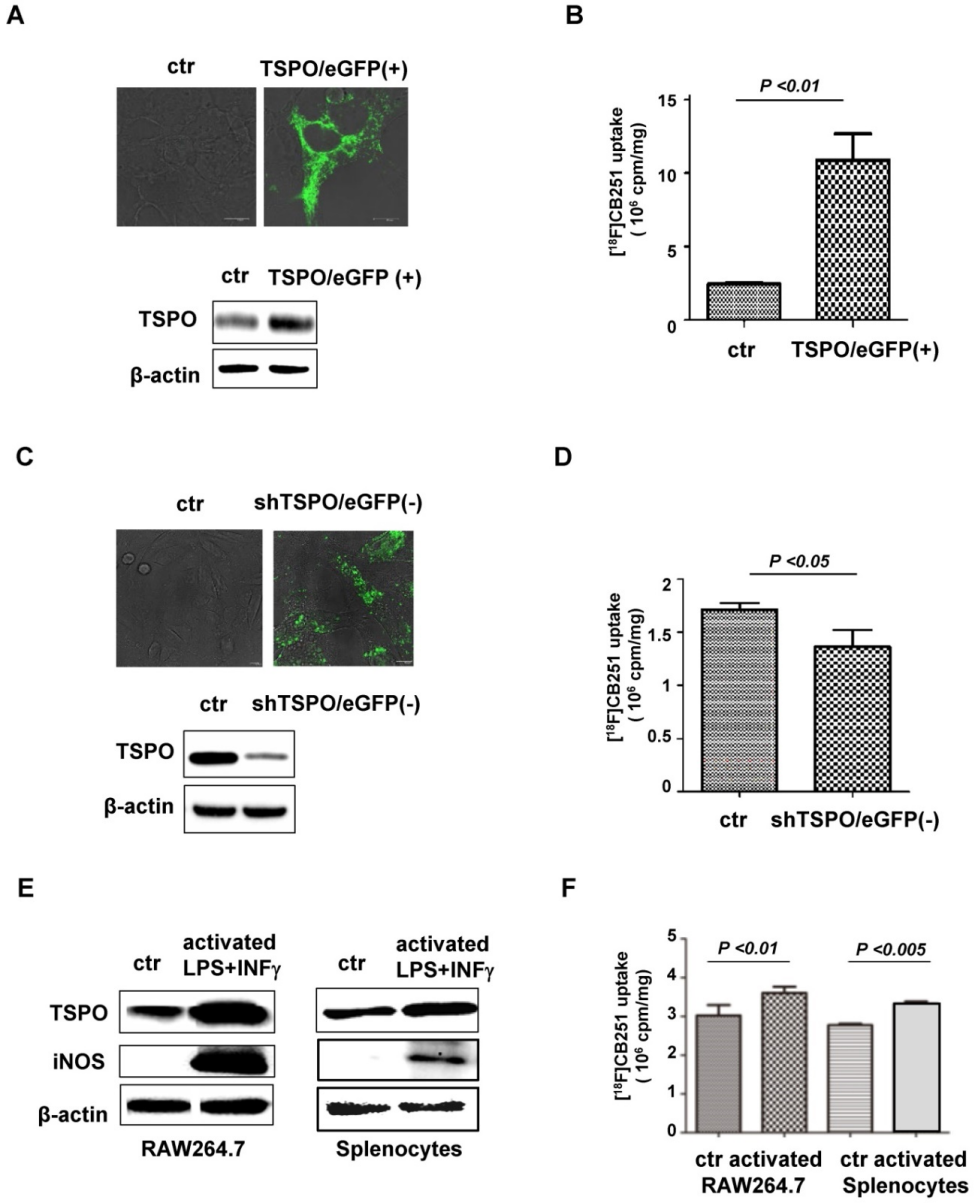
** [^18^F]CB251 as a TSPO-targeting ligand for imaging activated immune cells.** (**A**) TSPO expression in 293FT cells was increased by transfection with pCMV-TSPO/eGFP. (**B**) [^18^F]CB251 uptake was increased in TSPO-overexpressing cells. (**C**) TSPO expression in MDA-MB-231 cells was reduced by transfection with pCMV-shTSPO/eGFP. (**D**) [^18^F]CB251 uptake was decreased in cells with reduced TSPO expression. (**E**) Western blots of TSPO and iNOS (activated immune cell marker) in a mouse macrophage cell line (RAW264.7) and mouse splenocytes. (**F**) [^18^F]CB251 uptake was increased in activated cells (both in Raw264.7 and mouse splenocytes). All experiments were replicated at least three times.

In a previous study, the specificity of [^18^F]CB251 ligand for TSPO was not directly addressed in a biological system 26; therefore, in this study, the specificity of [^18^F]CB251 was tested by regulating TSPO expression in cell culture. [^18^F]CB251 uptake was measured in the cells with different levels of TSPO expression, which was regulated by transfection with pCMV-TSPO/eGFP for overexpression and pCMV-shTSPO/eGFP for reduced expression. 293FT cells expressing low levels of TSPO were used for TSPO overexpression (**Figure 2A**), and MDA-MD-231 cells expressing high levels of TSPO were used for reduced expression (**Figure 2C**). Fluorescence microscopic images showed increased eGFP signals, reflecting the successful transfection of each vector in the target cells (**Figure 2A-C**).

Increased TSPO expression was observed by both immunoblotting and fluorescence microscopy of pCMV-TSPO/eGFP transfected cells (**Figure 2A**), and [^18^F]CB251 uptake was increased in TSPO-overexpressing cells (**Figure 2B**). In contrast, decreased TSPO expression was observed in pCMV-shTSPO/eGFP-transfected cells (**Figure 2C**), and [^18^F]CB251 uptake was decreased in cells with reduced TSPO expression (**Figure 2D**). These data demonstrated that [^18^F]CB251 uptake was directly reflected by changes in TSPO expression, indicating that [^18^F]CB251 is specific for TSPO at the cellular level.

Next, we monitored TSPO expression as a marker for activated immune cells and tested whether [^18^F]CB251 could be used as a specific imaging probe for activated immune cells. The mouse macrophage cell line, RAW264.7, and primary white blood cells obtained from mouse spleens (splenocytes) were treated with LPS and IFN-γ for 24 h to activate immune cells. Following activation, immunoblotting in RAW264.7 cells and mouse splenocytes showed increased expression of TSPO as well as iNOS, a marker of activated macrophages (**Figure 2E**). Increased [^18^F]CB251 uptake was also observed in activated immune cells with increased TSPO expression (**Figure 2F**). These data indicated that [^18^F]CB251 directly reflected the activation of immune cells by targeting TSPO.

### BLI of peripheral immune cells in mice receiving an intracranial injection of LPS

In addition to activation of CNS immune cells, infiltration of peripheral immune cells through the BBB into the inflammatory area is considered to be critical for the neuroinflammatory process 34-36. Therefore, imaging depicting the infiltration of peripheral immune cells across the BBB provides useful information for the evaluation of the inflammatory process in the brain. Since BLI has been frequently used to visualize the distribution and proliferation of immune cells *in vivo* 37, 38, we employed this technique to observe peripheral immune cell infiltration into the neuroinflammatory region after systemic administration of luciferase-expressing peripheral immune cells. Splenocytes from luciferase-expressing transgenic mice (C57BL/6.Luc^Tg^) were used as a source of peripheral immune cells 38. We previously reported that the composition and function of immune cells in the C57BL/6.Luc^Tg^ mice were similar to those of the normal C57BL/6 mice 38.

**Figure 3:**
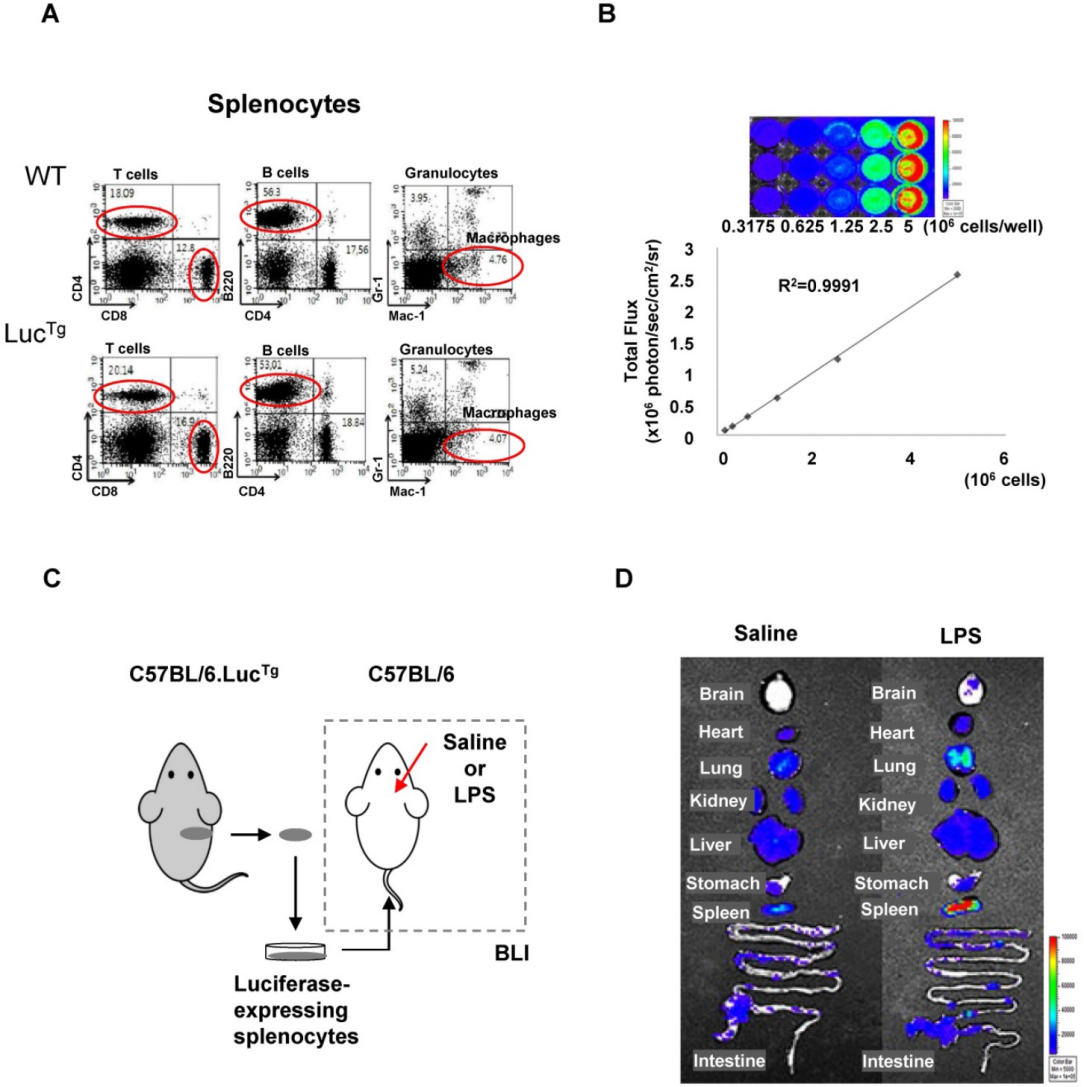
** Bioluminescence imaging of peripheral immune cells in a mouse neuroinflammation model using intracranial LPS injection.** (**A**) Flow cytometry analysis of immune cells from splenocytes of C57BL/6 (wild type) and C57BL/6.Luc^Tg^ (reporter transgenic) mice. (**B**) Semiquantitative analysis of luciferase-expressing mouse splenocytes from C57BL/6.Luc^Tg^. The intensity of luciferase signal and the number of cells were highly correlated (*R^2^*=0.9991) (**C**) Schematic representation of experimental design using luciferase-expressing splenocytes to monitor peripheral immune cell infiltration in the brain of a mouse with neuroinflammation induced by intracranial LPS injection. (**D**) Representative biodistribution of luciferase-expressing splenocytes in mice 4 days after intracranial injection of saline or LPS (N=2 for each treatment).

Flow cytometry analysis showed that splenocytes from reporter transgenic mice (C57BL/6.Luc^Tg^) used in this study had immune cell populations similar to those of normal (C57BL/6) mice (**Figure 3A**). The BLI signals of splenocytes isolated from luciferase-expressing transgenic mice increased in a cell-number-dependent manner with a high correlation value (*r^2^* = 0.9991), which could quantitatively assess the number of splenocytes that exhibited BLI signals (**Figure 3B**). To assess peripheral immune cell biodistribution by BLI, we isolated splenocytes from reporter mice and grafted them into inflamed mice (**Figure 3C**).

BLI signals produced by grafted luciferase-expressing splenocytes were observed in peripheral immune cell-targeting organs such as the lung, heart, liver, spleen, and intestine. However, BLI signals from grafted splenocytes were observed only in the brain of inflamed mice 4 days after intracranial LPS injection (**Figure 3D**). Most of the immune cell-homing organs showed bioluminescent signals in both saline-injected control mice and LPS-injected inflamed mice. However, the brain showed higher signals in mice receiving intracranial LPS injection than the saline injection. Since the mouse brain is not a homing organ of peripheral immune cells, our results indicated that grafted peripheral immune cells infiltrated the inflamed brain of mice.

### MRI using Gd-DOTA to monitor BBB disruption, immune cell infiltration, and activation

Our observations on peripheral immune cell infiltration of the LPS-injected mouse brains led us to monitor BBB disruption induced by mechanical stress. Our mouse model was created by injecting saline (control agent) or LPS (inflammation-inducing agent) into the mouse brain using a robotic stereotaxic device with a Hamilton syringe. We, therefore, had to investigate the possibility of peripheral immune cell infiltration through the injection hole rather than BBB. For this, closure of the injection hole and no BBB disruption in control mice had to be verified. Previously, Gd-DOTA (Dotarem), a gadolinium-based MRI contrast agent with a macrocyclic structure, has been used as an efficient paramagnetic contrast agent to increase MR sensitivity and specificity for the identification of BBB defects 39-41. Generally, T1-weighted MRI has been used to visualize the presence of gadolinium penetration through disrupted BBB. On the other hand, T2-weighted MRI usually provides information about water content or vasogenic edema of brain lesions induced by LPS injection. Therefore, we performed MRI using Gd-DOTA as a contrast agent to assess the level of BBB disruption and vasogenic edema in mouse models.

**Figure 4:**
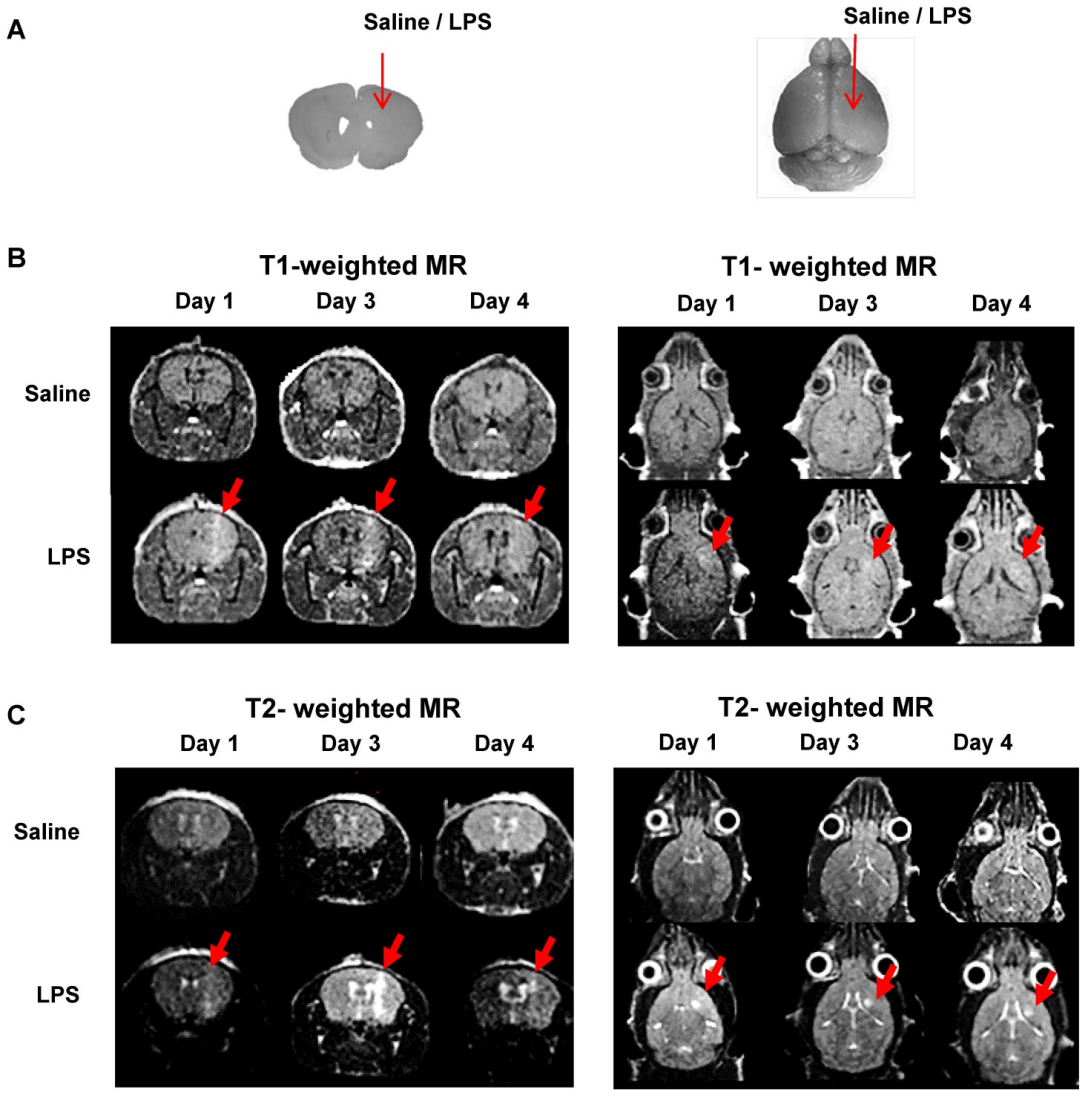
** Representative MR scans using gadolinium-DOTA to monitor the Blood-Brain Barrier (BBB) disruption and immune cell activation.** (**A**) Intracranial LPS injection model (saline as a control). (**B**) T1-weighted MR scans and (**C**) T2-weighted MR scans on day 1, 3, and 4 after intracranial injection (N=3). T1-weighted MRI was used to visualize the presence of gadolinium penetration through disrupted BBB, and T2-weighted MRI was used to monitor the vasogenic edema and localization of viable immune cells due to LPS induced-neuroinflammation.

We injected saline or LPS into the right striatum of the mouse brain to create intracranial inflammation (**Figure 4A**). The MR signals in the right striatum were higher in the LPS-injected mice than in the saline-injected mice in both the T1- (**Figure 4B**) and T2- weighted images (**Figure 4C**). Representative MR images demonstrated that BBB disruption caused by mechanical stress (injection hole) was recovered on day 1, as shown in the saline-injected group, while in the LPS-induced group, signal peaks indicating BBB disruption were still visible on day 3 (**Figure 4B**; N=3). As shown in **Figure 4C**, the T2 signal representing vasogenic edema due to LPS injection was observed. Peak T2 signal in LPS-treated mice, representing tissue damage resulting from inflammation, was observed on day 3. On day 4, T2 signals were reduced in the LPS-treated mice. Since T2-weighted signal enhancement was reported to be produced by gadolinium-based chelates trapped in viable immune cells 41, high T2 signals are thought to indicate the presence of viable immune cells in the region. Our results showed that tracer uptake on day 4 in T1- and T2-weighted MRIs with Gd-DOTA was due to the upregulation of neuroinflammation induced by LPS and not due to BBB disruption induced by mechanical stress. Based on these results, we used only T2-weighted MRI with Gd-DOTA to monitor the severity of neuroinflammation for further investigation.

### Simultaneous [^18^F]CB251 PET/MRI and BLI for visualization of neuroinflammation

**Figure 5:**
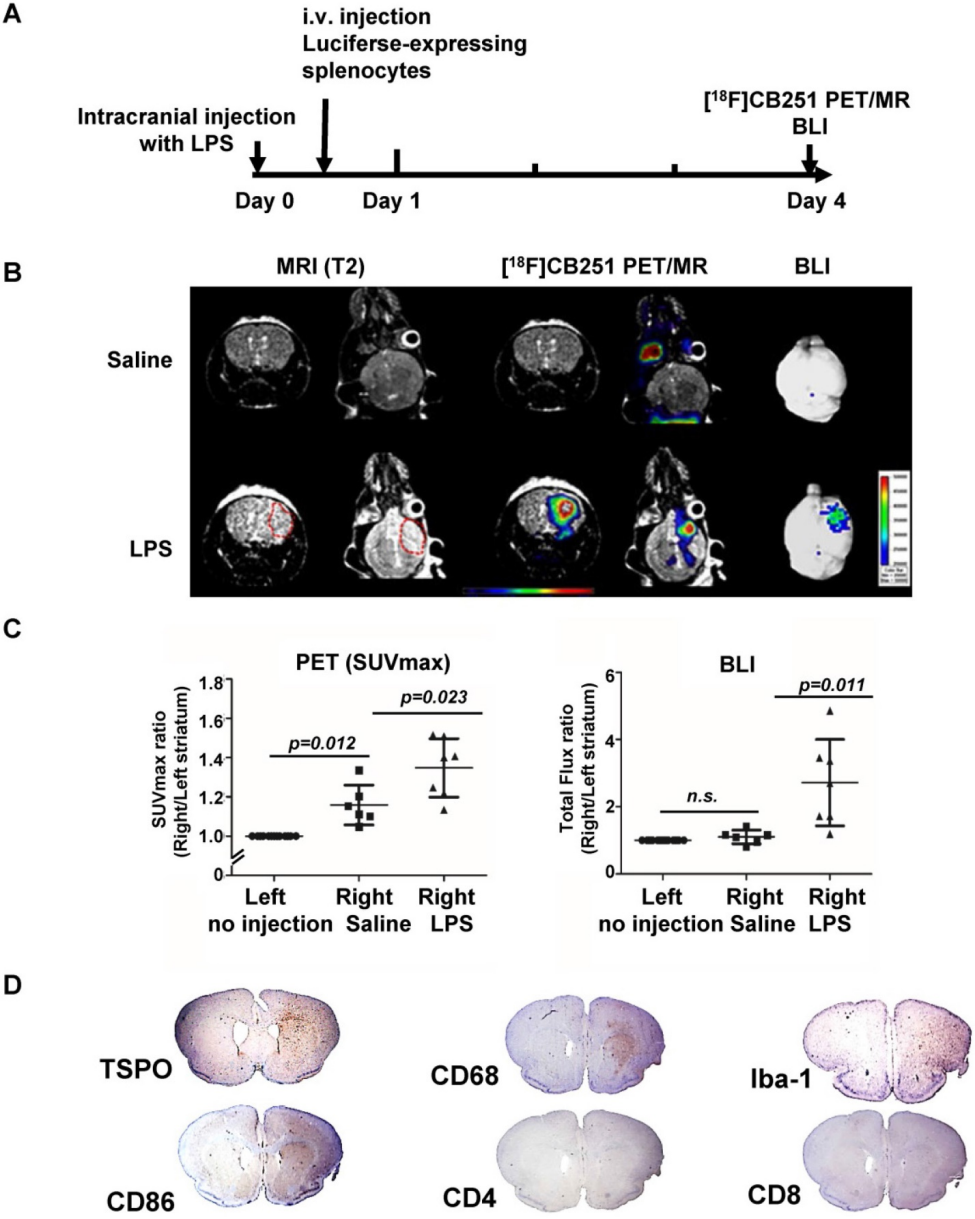
** Visualization of the intracranial LPS-induced neuro-inflammatory region using multi-modal imaging.** (**A**) Experimental scheme of simultaneous PET/MR using [^18^F]CB251 and BLI. (**B**) Representative images of [^18^F]CB251 PET/MR and bioluminescence (BLI). The red circle indicates a region of interest (ROI). (**C**) Signal intensity ratio of the right striatum (injected with saline or LPS) to the left striatum (no injection) in the saline-injected (N=6) and LPS-injected (N=7) mice. PET signal was expressed as standard uptake value (SUVmax) and the BLI signal was expressed as total flux (photon/sec/cm^2^/sr). (**D**) IHC staining of intracranial LPS injected mouse brain tissues. Iba-1 (macrophages/microglia marker), CD68 (macrophages/monocytes marker), CD86 (antigen-presenting cells), CD4 (helper T cells), and CD8 (cytotoxic T cells) were used as markers for each cell type.

It has been reported that simultaneous PET/MRI provides information on anatomical and functional changes with precise co-registration of signals and accurate correction of attenuation 42, 43. Herein, we performed simultaneous [^18^F]CB251 PET/MRI and BLI to visualize neuroinflammation indicative of immune cell activation and peripheral immune cell infiltration in brain regions in the intracranial LPS mouse model (**Figure 5A**). Although T1- and T2-weighted MR signals peaked on day 3 after LPS injection, neuroinflammation was still observed on day 4. Imaging studies at this time point could provide additional useful information about peripheral immune cell infiltration without interference due to severe tissue damage. Therefore, we performed [^18^F]CB251 PET/MRI and BLIs on day 4.

Radiation signals from [^18^F]CB251-targeting TSPO proteins reflected activation of immune cells, such as resident microglia and infiltrated peripheral immune cells. T2-weighted MR signals reflected localization of all viable immune cells, and the BLI signals reflected only luciferase-expressing peripheral immune cells. On day 4, LPS-injected mice showed higher signals from each imaging modality in the inflamed region of the right striatum than in the saline-injected mice (**Figure 5B-C**). T2-weighted MR signals from LPS-injected mice appeared to emanate from a slightly wider area of immune cells (**Figure 5B left**) than the [^18^F]CB251 PET signals (**Figure 5B middle**), but the critical region of T2-weighted MR signal was co-localized with the region of increased [^18^F]CB251 PET signals. In the BLI, luciferase-expressing splenocytes representing peripheral immune cells were visualized in the right striatum of the LPS-injected mice (**Figure 5B right**). The degree of neuroinflammation could be observed using T2-weighted MR reflecting localization of all viable immune cells, including residential and peripheral immune cells. However, we used MR images of simultaneous PET/MR to obtain anatomical information for locating inflammation and BBB disruption, since the observation of inflammatory cell activation using quantitative PET is more sensitive.

Next, we compared the differences between the signal intensity ratio of the right (saline or LPS injection) to the left (no injection) striatum in the saline- (N=6) and LPS-injected (N=7) mice (**Figure 5C**). [^18^F]CB251 PET signal was expressed as standard uptake value (SUVmax), and BLI optical signal was expressed as total flux (photon/sec/cm^2^/sr). In LPS-injected mice, [^18^F]CB251 radioactivity and BLI optical signals were significantly increased compared to saline-injected mice (*p*=0.023 for [^18^F]CB251;* p*=0.011 for BLI ). When we compared the difference between the non-injected left striatum and the saline-injected right striatum, [^18^F]CB251 PET signal increased slightly in the saline-treated right striatum (*p*=0.012). However, the BLI signal did not change significantly in the saline-treated right striatum. Since [^18^F]CB251 PET reflects residential microglial activity as well as peripheral immune cell activity, these results suggested that [^18^F]CB251 PET was more sensitive to mechanical stress due to intracranial injection than BLI, which only reflected peripheral immune cells. Considering the change in the strength of BLI signals after LPS injection, BLI appeared to be more sensitive to the development of inflammation than PET/MRI using the TSPO-targeting probe [^18^F]CB251. However, the BLI signals were not detectable through the black skin of mice, which had to be removed to detect and assess the BLI signals. Taken together, the simultaneous analysis by [^18^F]CB251 PET targeting TSPO and BLI targeting grafted peripheral immune cells revealed resident and peripheral immune cell activation due to inflammatory processes, as well as the location of infiltrating peripheral immune cells.

### Identification of immune cells in inflamed regions of the mouse brain

We performed immunohistochemical (IHC) staining to identify the presence of different types of immune cells in the brain region of neuroinflammation (**Figure 5D**). Antibodies were used for staining TSPO-expressing cells (anti-TSPO) and immune cells in the CNS parenchyma including perivascular macrophages, monocytes, microglia (anti-CD68, anti-Iba-1), antigen-presenting cells (anti-CD86), and T cells (anti- CD4, CD8). Cells from monocyte lineages such as circulating macrophages and resident microglia expressing CD68 were clearly increased and were located with TSPO expressing cells at the site of inflammation (right striatum). Also, the antigen-presenting cells expressing CD86, which are expressed only in peripheral immune cells and critical for T cell activation, increased significantly in the right striatum of LPS-injected mice. Since macrophages and dendritic cells from bone marrow generally exhibit higher antigen-presenting ability than microglial cells, we confirmed that infiltrated peripheral immune cells were localized at the site of inflammation. However, helper T cells (anti-CD4) and cytotoxic T cells (anti-CD8) appeared widely distributed on the cross-sections of mouse brains.

Animals were sacrificed 4 days after LPS injections, and only the initial response consisting of activated resident immune cells and recruited antigen-presenting cells could be observed at the sites of inflammation. Our multimodality imaging data and IHC data colocalizing TSPO-, CD68- and CD86-expressing cells in the inflamed region of the brain, clearly demonstrated that immune cells in the LPS-injected region were activated, and recruitment of peripheral immune cells occurred after intracranial LPS challenges.

### Comparative analysis of neuroinflammation by [^18^F]CB251 PET and BLI after anti-inflammatory treatment

**Figure 6:**
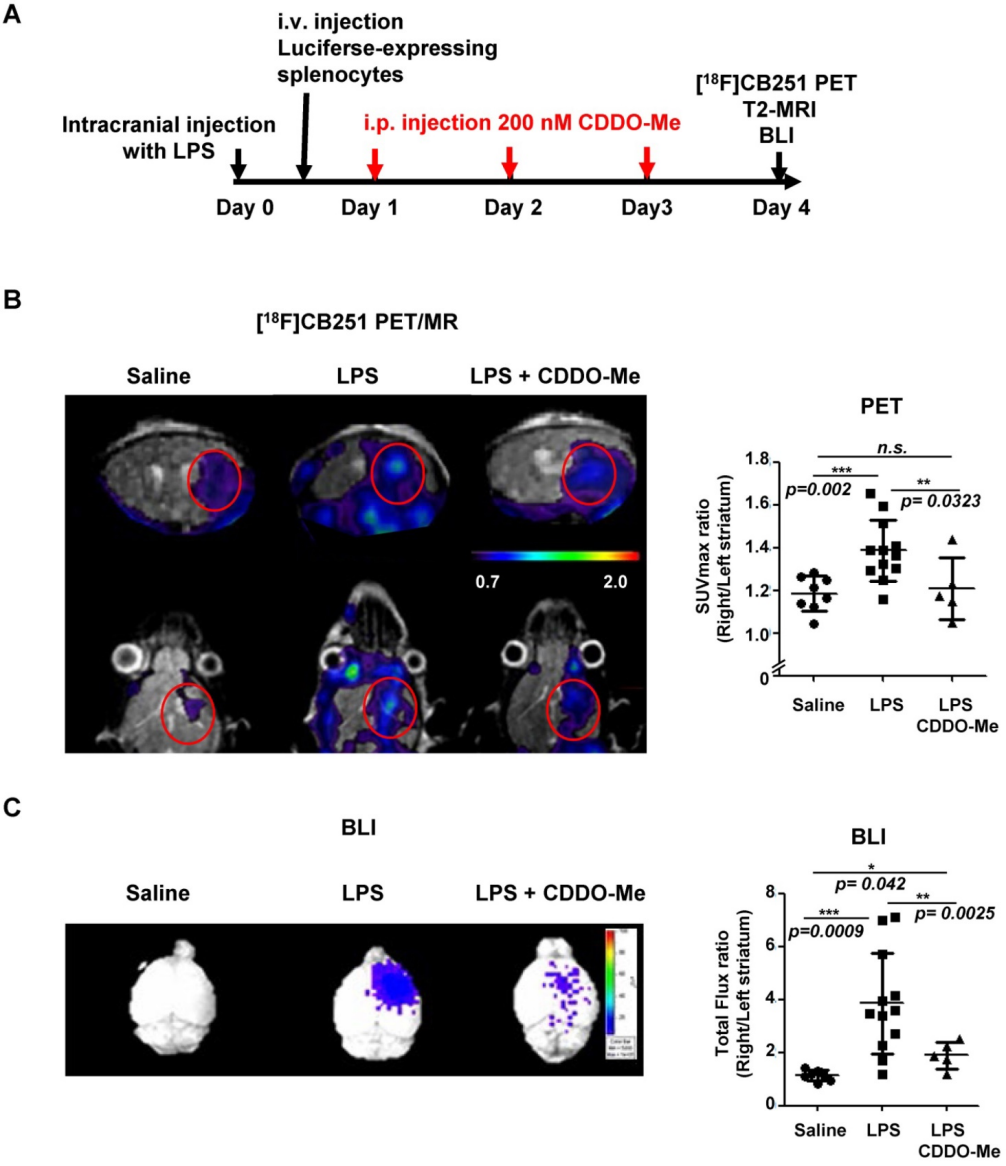
** Evaluation of the therapeutic effect of the anti-inflammatory drug (CDDO-Me) using [^18^F]CB251 PET and BLI.** (**A**) Experimental design of treatments and imaging schedules, including simultaneous [^18^F]CB251 PET/MRs and BLIs. (**B**) Representative [^18^F]CB251 PET/MR scans and SUVmax ratios were plotted. (**C**) Representative BLI image and total flux ratio were plotted. Signal ratios from each modality (SUVmax ratio for PET; Total flux ratio for BLI) were calculated from the region of interest (ROI) of right (intracranially injected with saline, LPS, or LPS plus anti-inflammatory agent CDDO-Me) and left striatum (non-injected) in the mouse brain. The values are mean ± SD. (N=8 for saline-treated; N=12 for LPS-treated; N=5 for LPS plus CDDO-Me).

We compared the usefulness of [^18^F]CB251 PET with BLI to evaluate the therapeutic efficacy of an anti-inflammatory agent in neuroinflammation. We used 2-cyano-3, 12-dioxooleana-1, 9-dien-28-oic acid methyl ester (CDDO-Me), which is known to inhibit inflammatory cytokines such as IL-6, IL-10, and IL-12 44-48. We administered CDDO-Me daily for 3 days after intracranial LPS-injection in the right striatum (**Figure 6A**). As shown in the previous experiments, the [^18^F]CB251 uptake ratio (SUVmax, right/left striatum) of the inflamed region in LPS-injected mice was significantly increased compared to saline-injected control mice (*p*=0.002). On the other hand, the [^18^F]CB251 uptake ratio of CDDO-Me-treated mice after LPS injection significantly decreased compared to LPS-injected mice (*p*=0.0323, **Figure 6B**). A similar pattern was also confirmed by the ratios of SUVmean values (data not shown).

In LPS-injected mice, the BLI signal ratio from the brain (expressed as the ratio of the total photon flux of the right and the left striatum) was 3.93-fold higher than that from saline-injected control mice (*p*=0.0009). The BLI signal ratio from the brain of mice treated with anti-inflammatory CDDO-Me after LPS injection decreased dramatically compared to LPS-injected mice (*p*=0.0025, **Figure 6C**). These data demonstrated that CDDO-Me successfully inhibited peripheral immune cell infiltration and reduced their recruitment leading to neuronal damage. Our results clearly showed that both modalities, TSPO-targeting [^18^F]CB251 PET and peripheral immune cell-targeting BLI, revealed the degree of neuronal inflammation and reduced activation of immune cells.

## Discussion

In a previous report 26, we described synthesis, binding, and competition studies with [^18^F]CB251 and its future applications. In this study, we attempted to test [^18^F]CB251, targeting TSPO as a clinically applicable PET probe for imaging neuroinflammation. Our results indicated that [^18^F]CB251 targeted TSPO with higher selectivity in human TSPO polymorphisms (**Figure 1**), was specific for TSPO protein, and taken up by activated immune cells with high TSPO expression (**Figure 2**), These observations suggested that [^18^F]CB251 is a suitable candidate for imaging of TSPO-related neuroinflammation. We also established an intracranial LPS-induced neuroinflammation mouse model, and designed experiments with a multimodal imaging approach using [^18^F]CB251 PET/MR and BLI technologies to understand the interaction between CNS and the peripheral immune system during neuroinflammation. Furthermore, we tested whether [^18^F]CB251 PET could be used to evaluate the therapeutic effect of anti-inflammatory drugs in this model.

The higher affinity of a ligand for the target protein does not automatically reflect the target selectivity. Especially, *in vitro* and *in vivo* discrepancies of targeting ability of TSPO ligands have been reported in many human brain studies 20-25, 27, 28, 33, 49, 50. While developing TSPO targeting ligands, we noticed that human rs6971 polymorphism was important, and TSPO ligand binding was influenced by A147T mutation (**Figure S1A**). Generally, TSPO with Ala at 147th amino acid is the abundant phenotype and high-affinity ligand binder (HAB). Single point mutation of C to T in the TSPO gene induces amino acid change (Ala to Thr at 147th amino acid). This phenotype is a low-affinity ligand binder (LAB). PBR28 is a well-known TSPO ligand that shows a different binding ability to the TSPO rs6971 polymorphism.

Although testing the sensitivity of the rs6971 polymorphism (A147T) for developing clinically applicable TSPO targeting ligands is important, we could not find enough donors with the rs6971 polymorphism to test the affinity for [^18^F]CB251 in Korea. About 30% of Caucasian and 3% of Chinese/Japanese are reported as LABs (“TSPO polymorphism by ethnic differences” in the Hapmap database http://hapmap.ncbi.nih.gov or http://asia.ensembl.org/Homo_sapiens/Variation/Explore?r=22:43162420-43163420;v=rs6971;vdb=variation;vf=210028659). The LAB percentage of Koreans has not been reported, but less than 3% of Koreans are expected to have LAB considering the genetic similarity between the Chinese and Japanese.

Thus, we established cells that ectopically express polymorphic TSPOs (WT for HAB or Mut-A147T for LAB). *In vitro* site mutagenesis was introduced in the coding sequence of 147th Ala in TSPO (**Figure S1B**). To test the sensitivity of the rs6971 polymorphism, competitive inhibition assays of CB251 and other ligands with [^3^H]PK11195 were performed (**Figure 1B**). We calculated the IC_50_ value of each ligand to [^3^H]PK11195 from % inhibition in HAB or LAB. The ratios of IC_50_ LAB/HAB for each ligand were also calculated. The ratio of IC_50_ values of PK11195 was consistent with previous reports (0.83 to 0.85 for PK11195 34, 35). Similar to PBR28, the ratios of IC_50_ LAB/HAB for fm-PBR28-*d_2_* was 37.28 (55 for PBR28 34, 35), indicating lower binding to LAB. The ratios of IC_50_ LAB/HAB were 1.14 for CB251 and 3.96 for GE-180 33. After comparing the Ki (generated from recombinant TSPO protein) and the ratio of IC_50_ LAB/HAB (generated from membrane TSPO from cells), we concluded that CB251 had a higher binding affinity (Ki=0.27 nM) and selectivity (ratio of IC_50_ LAB/HAB=1.14) than other TSPO ligands regardless of TSPO polymorphism.

To test *in vitro* and *in vivo* selectivity for rs6971 polymorphism of [^18^F]CB251 as a PET probe, [^18^F]CB251 cell uptake PET scans were performed. Our results showed that [^18^F]CB251 had a higher binding affinity (**Figure 1A**) with favorable selectivity (**Figure 1B-E**) *in vitro* and *in vivo*. However, there were limitations because cellular uptake of TSPO ligands in humans can be influenced by factors such as cellular metabolism, endogenous TSPO level, and *in vivo* kinetics. [^11^C]PK11195 and [^11^C]ER176 were both reported to be significantly insensitive to TSPO polymorphism *in vitro*, however, in human studies, [^11^C]PK11195 showed the different uptake of lung and heart in binders and non-binders 49. In addition, [^11^C]ER176 represented different radioactivity according to each genotype in the human brain 50. Therefore, it is difficult to conclude that *in vitro* uptake completely reflects human kinetics. Unfortunately, we could not perform human study because it is difficult to find Korean volunteers to express rs6971 A147T polymorphism. Evaluation of the ligand in humans should be performed as a further study.

In this study, we also tested the specificity of [^18^F]CB251 as a TSPO-targeting ligand for imaging activated immune cells (**Figure 2**). By modulating the TSPO expression levels in the cells and immune cell activation, we found that [^18^F]CB251 was specific for TSPO protein and its uptake was specifically observed in activated immune cells with high TSPO expression.

The role of resident immune cells in the brain and peripheral immune cells that infiltrate across the BBB in neuroinflammation, have been studied and emphasized 34-36 as the main targets of the neuroinflammation imaging. The resident CNS immune system usually consists of innate immune cells and operates under homeostatic conditions. However, when peripheral innate and adaptive immune cells enter the CNS, they execute distinct cell-mediated effects. Infiltration of peripheral immune cells across the BBB exacerbates neuroinflammation resulting in higher levels of neurotoxicity 4, 8. Therefore, it is important to visualize peripheral immune cell infiltration to evaluate neuroinflammatory damages. Herein, we tested whether [^18^F]CB251 PET signal reflects neuroinflammation compared to other conventional methods such as MRI and BLI.

We adoptively transferred splenocytes from luciferase-expressing transgenic mice into an intracranial LPS-injected mouse model to visualize the distribution of infiltrating peripheral immune cells in the brain by BLI. The total population of splenocytes was used because immune responses are initiated by the co-stimulation of different populations of immune cells. Despite the limitations of optical imaging, the grafted splenocytes were found in the inflamed region of the mouse brain (**Figure 3**).

We also visualized BBB disruption and immune cell localization by MRI with the contrast agent, Gd-DOTA. When Gd-DOTA crosses the damaged BBB into the extracellular space of brain parenchyma, its paramagnetic property is changed 41, 51. T1-weighted MRI contrast enhancement shows the rapid diffusion of gadolinium-based chelates from non-viable cells, while T2-weighted MRI contrast enhancement shows slow diffusion of gadolinium-based chelates from viable immune cells 39. We used T1-weighted MRI to visualize gadolinium penetration through disrupted BBB. T2-weighted MRI was used to monitor the vasogenic edema and localization of viable immune cells, including residential microglia and peripheral immune cells due to LPS induced-neuroinflammation (**Figure 4**). In our mouse model of neuroinflammation induced by intracranial injection of LPS, we opted to use T2-weighted imaging. However, since the sensitivity to detect BBB disruption is limited, there is a limitation that only MRI signal is not sufficient to evaluate BBB disruption. Therefore, it is difficult to exclude the possibility of micro-disruptions of BBB.

To validate the usefulness of [^18^F]CB251 as a PET probe for imaging neuroinflammation, we performed simultaneous PET/MRI and BLI (**Figure 5**), which provided information on anatomical and functional changes with precise co-registration of signals and accurate correction of attenuation 42, 43. IHC staining showed increased TSPO expression at the site of neuroinflammation. It also demonstrated that inflammatory monocytes, macrophages, and antigen-presenting cells were present with resident immune cells (i.e., microglia) at the site of inflammation, suggesting cooperation between the peripheral and CNS immune system in the region of neuroinflammation 34-36. BLI revealed peripheral immune cell infiltration only in the region of the inflamed brain, but the radioactive signal from [^18^F]CB251 showed the effects of both infiltrating peripheral immune cells and resident microglia.

Furthermore, simultaneous [^18^F]CB251 PET/MR and comparative analysis of [^18^F]CB251 PET and BLI were performed to evaluate the efficacy of an anti-inflammatory agent and verify the usefulness of [^18^F]CB251 PET. CDDO-Me was used as an anti-inflammatory drug, which was reported to induce antioxidant proteins that prevent oxidative damage from inflammation 44-48. In our LPS-induced neuroinflammation model, treatment with CDDO-Me reduced [^18^F]CB251 PET and BLI signals, indicating a decrease in inflammatory response and recruitment of peripheral immune cells (**Figure 6**). Despite the higher sensitivity of BLI, its signals were obscured by the skin and black fur of the mice in our animal model. Because of the limitations of optical imaging, BLI can be used only for preclinical studies. However, [^18^F]CB251 PET is a noninvasive imaging procedure that is directly applicable to clinical investigations that provide diagnostic and therapeutic information on neuroinflammation.

We demonstrated that [^18^F]CB251 is specific to TSPO-expressing inflammatory cells with better selectivity for TSPO polymorphism. TSPO-targeting [^18^F]CB251 PET identified the regions of neuroinflammation and also allowed us to evaluate the therapeutic effect of anti-inflammatory treatments. In preclinical studies, our multimodal imaging approach using PET/MR and BLI is expected to enable further understanding of the interactions between CNS and the peripheral immune system during neuropathogenesis. Furthermore, [^18^F]CB251 PET/MRI, which provides information on anatomical and functional changes with precise co-registration of signals, can be an efficient tool for testing the efficacy of various anti-inflammatory drugs and treatment planning for both preclinical and clinical application.

## Materials & Methods

### Cell culture and reagents

RAW264.7, a mouse macrophage cell line and mouse splenocytes were used to evaluate TSPO expression. LPS (50 ng/mL, Sigma-Aldrich, St.Louis, MO, USA) and IFN-γ (20 ng/mL, Sigma-Aldrich) were used to activate immune cells. The 293FT (Invitrogen, Carlsbad, CA, USA) and MDA-MB-231 (ATCC, Manassas, VA, USA) cell lines were used for various TSPO expression levels. Cells were maintained in complete DMEM (for Raw264.7 and MDA-MB-231) or MEM (for 293FT) with 10% FBS and 1% antibiotics (penicillin-streptomycin). All media and reagents for cell culture, including FBS and antibiotics were purchased from Gibco (Grand Island, NY, USA).

### TSPO expression in Cells

To express the genetic variants of TSPO proteins (Wild-type, WT; Mutant with alanine-threonine at 147 amino acid, A147T), we generated different TSPO expression vectors from the PBR (TSPO) (NM_000714) human tagged ORF clone RG220107 (Origene, Rockville, MD, USA) by site mutagenesis (Figure S1B). We changed the nucleotide sequences encoding alanine at amino acid 147 to threonine (A147T). We then transfected these vectors into 293FT cells and performed an *in vitro* uptake test of these cells at 24 h after transfection.

For overexpression of the TSPO protein, the 293FT cell line was transiently transfected with vectors (pCMV-eGFP/TSPO) (AddGene, Cambridge, MA, USA) harboring the eGFP and TSPO coding sequences. MDA-MB-231, which highly expresses TSPO, was used for the reduction of TSPO expression by shRNA (pCMV-eGFP/shTSPO; AddGene). Each vector used for various levels of TSPO expression was transfected by Lipofectamine 2000 (Invitrogen, Waltham, MA, USA) at a total concentration of 2.5 µg/mL. Fluorescent images were acquired by a confocal laser-scanning microscope (Leica TCS SP8; Wetzlar, Hesse, Germany), and Western blotting was performed for verification.

### Western blotting

Cell lysates were extracted by RIPA lysis buffer (Sigma-Aldrich), with a protease inhibitor (Roche, Branchburg, NJ, USA) cocktail. Protein concentrations were measured by the BCA protein assay kit (Pierce Endogen, Rockford, IL, USA), and samples containing 20 µg of proteins were separated on 10% polyacrylamide gels. Proteins were then transferred to PVDF membranes (Millipore, Watford, Herts, UK), which were blocked by 5% skim milk in Tris-buffered saline (20 mM Tris, 138 mM NaCl, and pH 7.4) with Tween-20 (0.1%) for 1 h. Membranes were then incubated with primary antibodies followed by incubation with secondary antibodies. The following antibodies were used: anti-TSPO (Abcam, Cambridge, Cambs, UK), anti-iNOS (Santa-Cruz Biotechnology, Santa-Cruz, CA, USA), anti-β-actin (Sigma-Aldrich) were anti-rabbit IgG (Cell Signaling Technology, Danvers, MA, USA), and anti-mouse IgG (Cell Signaling Technology) for β-actin.

### Radiochemical synthesis of radioactive TSPO-targeting ligands

The [^11^C]PK11195 21, deuterium-substituted fluoromethyl-PBR28 ([^18^F]fmPBR28-*d_2_* 52), and [^18^F]GE-180 33 were synthesized according to previous studies. The [^18^F]CB251 probe was also synthesized following a protocol described in a previous study 26.

### Membrane protein extraction

Membrane protein extraction was conducted by protocols from the membrane protein extraction kit (Mem-PER plus kit, Thermo Fisher, Waltham, MA, USA). 293FT cells (5 × 106/100 Ø) were seeded for transient transfection and transfected with TSPO WT or A147T vector for 24 h. Cells were harvested using a scraper and washed twice with cell wash solution. After centrifugation (300 × g, 5 min), the cell pellet was permeabilized for 10 min at 4°C and centrifuged (16000 × g, 15 min, 4°C). The supernatant containing cytosolic proteins was removed, and the pellet was solubilized with the buffer from the Mem-PER plus kit for 30 min at 4°C. The solubilized pellet was centrifuged again (16000 × g, 15 min, 4°C), and the supernatant containing membrane-associated proteins was used for competitive binding assay.

### Competitive inhibition assay

To verify the binding affinity of TSPO-targeting ligand for the TSPO polymorphism rs6971, we performed competitive inhibition assay 34, 35 with [^3^H]PK11195 (NET885250UC, 9.25 MBq, Perkin Elmer, Boston, MA, USA) and 3 different TSPO-targeting ligands (PK11195, fmPBR28-*d2*, and CB251). Membrane proteins (100 µg/100 µL) isolated from 293FT cells expressing TSPO WT (HABs) or A147T (LABs) were incubated with [^3^H]PK11195 (0.019 MBq/100 µL) at different concentrations of each ligand (from 0.1 nM to 1000 µM) in assay buffer (50 mM Tris base, 140 mM NaCl, 1.5 mM MgCl_2_, 5 mM KCl, 1.5 mM CaCl_2_, pH 7.4) for 1 h at 37°C. Subsequently, the mixtures were filtrated through Whatman filter paper under vacuum and washed three times with the wash buffer (50 mM Tris base, 1 mM MgCl_2_, pH7.4). Filters were harvested, and the radioactivity of each filter in scintillation buffer (Perkin Elmer) was counted using β-counter (Liquid scintillation analyzer Tri-Carb3100 TR, Perkin Elmer, Waltham, MA, USA). IC_50_ values of each ligand to [^3^H]PK11195 in HAB or LAB were measured. The ratio of IC_50_ values of each ligand to [^3^H]PK11195 in the LAB to HAB was also calculated, representing the sensitivity of TSPO polymorphism. A value close to 1 indicates lower sensitivity to TSPO polymorphism.

### Measurement of cell uptake of radioactive ligands

Cells were seeded in 6-well plates (Sigma-Aldrich) until they reached approximately 80% confluency. Before the uptake experiments, the cells were preincubated with glucose-free RPMI-1640 medium (Gibco) for 4 h. The cells were then trypsinized (Gibco), counted, and 1×10^5^ cells transferred into a 5 mL test tube containing warmed Hank's balanced salt solution (HBSS, Sigma-Aldrich) with 0.5% (w/v) bovine serum albumin (Sigma-Aldrich). Subsequently, approximately 0.185 MBq of radioactive ligands were added to the tubes, and the cells were incubated in a humidified incubator with 5% CO_2_ for 1 h at 37°C. The cells were then washed 3 times with cold HBSS and lysed for 5 min in 200 μL of 1% sodium dodecyl sulfate (SDS, Sigma-Aldrich). Cell lysates were collected, and radioactivity was measured by a Cobra II gamma counter (Canberra Packard; Vaughan, Ontario, Canada). Radioactivity was normalized according to the amount of total protein at the time of assay. Total protein levels were quantified by the BCA protein assay kit (Pierce, Rockford, IL, USA). All experiments were performed in triplicate.

### Preparation of splenocytes from B6. Luc^Tg^ mice

Luciferase-expressing cells were isolated from the dissected spleens of transgenic C57BL/6luc^Tg^ mice and centrifuged at 1300 rpm for 5 min at 4 °C). The red blood cell lysis buffer (1 mL/spleen, Sigma-Aldrich) was added to the cell pellet, and the mixture was incubated at room temperature for 3 min to remove red cells. After a double volume of RPMI medium was added to the cells, 2-3 volumes of red blood cell lysis buffer were added until complete lysis of red blood cells. Subsequently, the cells were re-suspended, ensuring a suspension of single cells, and filtered through a cell strainer (40 µm nylon, Falcon, Oneonta, NY). Cells (3.175 × 10^5^ to 5 × 10^6^) were then plated in 24-well plates, and bioluminescent images were acquired after the addition of D-luciferin (300 μg/mL, Caliper Life Science, Hopkinton, MA) to measure luciferase activity. Bioluminescence intensity was measured by an IVIS 100 system (Perkin Elmer, Waltham, MA). Cells (2 × 10^7^) were adoptively transferred by intravenous injection into recipient C57BL/6 mice to monitor the *in vivo* distribution of luciferase-expressing immune cells. All experimental designs and procedures for animal experiments were approved by the Institutional Animal Care and Use Committee of Seoul National University and in accordance with the ethical standards of Seoul National University Hospital guidelines of Seoul National University Hospital.

### Intracranial inflammation mouse model by LPS administration

C57BL/6 mice (male, 6 weeks old) were used for generating the intracranial inflammation model. LPS (Sigma-Aldrich, 5 µg/2 µL dissolved in saline) was used to induce local inflammation in the right striatum of the mouse brain (anteroposterior, +1.8; mediolateral, +2.0; dorsoventral, -3.0) via intracranial injection by a robotic stereotaxic device (Stoelting, Wood Dale, IL) equipped with a Hamilton syringe at a rate of 0.25 µL/min 52-54. Saline was injected as a control. The mouse was placed in the isoflurane induction box (5% isoflurane, 95% air, 250 cc/min) until the animal was fully anesthetized, and the isoflurane percentage was adjusted to 2% at a flow rate of 250 cc/min during the process.

### [^18^F]CB251 PET/MR imaging

To visualize neuroinflammation, PET/MR scans were acquired by a simultaneous small animal PET insert (SimPET, Brightonic imaging, Seoul, Korea) combined with an M7 1.0 T MRI system (Aspect Imaging, Jerusalem, Israel). [^18^F]CB251 (9.25 to 11.1 MBq per mouse) was injected intravenously, and images were acquired 4 days after intracranial injection of LPS. Mice were anesthetized with 1.5% isoflurane during imaging.

To evaluate breach of the BBB, gadolinium (Gd-DOTA, 0.5 mmol/kg) was injected intravenously, and T1- and T2-weighted MR scans were acquired 26. The T1-weighted fast-spin echo sequence consisted of a repetition time of 200 msec and an echo time of 10 msec. The T2-weighted fast spin echo sequence consisted of a repetition time of 3000 msec and an echo time of 63.84 msec.

A PET/MR scan was performed simultaneously for 40 min with a 30 x 30 mm field-of-view (FOV) at 20 min post-[^18^F]CB251 injection. PET images were reconstructed using 3D OSEM algorithm (12 subsets and 3 iterations). An ellipsoidal region-of-interest was drawn around the peak PET activity on the right striatum in a coronal PET/MRI slice, and the region-of-interest was copied to the left striatum using AMIDE program (ver 0.9.0, http://amide.sourceforge.net). PET and MR images were reconstructed and analyzed by the AMIDE program (ver 0.9.0, http://amide.sourceforge.net). The ratio of maximum PET SUV in the right and left striatum was then calculated.

### Bioluminescence imaging

An *in vivo* IVIS 100 bioluminescence/optical imaging system (Xenogen, Alameda, CA, USA) was used to follow luciferase-expressing peripheral immune cells. For the *ex vivo* imaging of luciferase-expressing peripheral immune cells in the brain, D-luciferin (3 mg/mouse) was intravenously injected into the tail vein at 10 min before sacrificing the animal in a CO_2_ chamber. The bioluminescent signal was acquired and analyzed by Living Image ver.2.50.2 software (Xenogen).

### Immunohistochemistry

Brain tissues from mice were fixed in 4% paraformaldehyde, embedded in paraffin, and sectioned into 4 µm-thick segments. After heat-induced antigen epitope retrieval, slides were blocked with PBS containing 3% normal serum and incubated overnight at 4 °C with primary antibodies as follows: anti-CD68, anti-CD86 (Santa Cruz Biotechnology, Santa Cruz, CA, USA), anti-CD4, anti-CD8, anti-Iba-1, and anti-TSPO (Abcam, Cambridge, UK). The slides were then washed with PBS and incubated with biotinylated secondary antibodies (anti-goat for CD68; anti-rabbit for CD4, CD8, and Iba-1; all from Santa Cruz Biotechnology). The slides were then treated with avidin-biotin solution (Vector Laboratories, Burlingame, CA, USA) for 1 h. The antigenic signal was developed using DAB (3, 3 -diaminobenzidine) substrate (Vector Laboratories, Burlingame, CA, USA), according to the manufacturer's instructions. Finally, the slides were counterstained with hematoxylin and mounted with the Permount Mounting Medium (Thermo Fisher Scientific, Fair Lawn, NJ, USA).

### CDDO-methyl ester (CDDO-Me) treatment

2-Cyano-3,12-dioxo-oleana-1,9(11)-dien-28-oic acid methyl ester (CDDO-methyl ester or CDDO-Me, Sigma-Aldrich) was dissolved in DMSO to produce a 10 mM stock solution. The stock solution was further diluted with 7.5% PBST (1.5 ml Tween-20 dissolved in 20 ml PBS) to obtain a 200 nM CDDO-ME solution. Mice received 100 µL of CDDO-ME solution via intraperitoneal injection administered once daily for 3 days beginning 1 day after intracranial administration of LPS.

### Statistical analysis

All results were calculated as means ± standard deviation (SD). Statistically significant differences were analyzed by a paired 2-sample Student t-test. Statistical significance was considered to be *p*<0.05. GraphPad Prism 5 software (San Diego, CA) was used for statistical analysis.

## Supplementary Material

Supplementary figures and tables.
