# Supplementary material for: [18F]CB251 PET/MR imaging probe targeting translocator protein (TSPO) independent of its Polymorphism in a Neuroinflammation Model
Source: Theranostics. 2020 Jul 23;10(20):9315–31. doi: 10.7150/thno.46875 (PMC7415805; doi:10.7150/thno.46875)
Supplement: Supplementary file 1 — Supplementary figures and tables. [file thnov10p9315s1.pdf]

A

TSPO ligand binding classification

TSPO genotype			Ligand Binding Phenotype
DNA polymorphism rs6971		Protein position 147	
Wild type	C/C	Ala/Ala	HAB*
Mutant	T/T	Thr/Thr	LAB*

HAB High Affinity Binder ; LAB Low Affinity Binder

B

Vectors for TSPO Mutagenesis

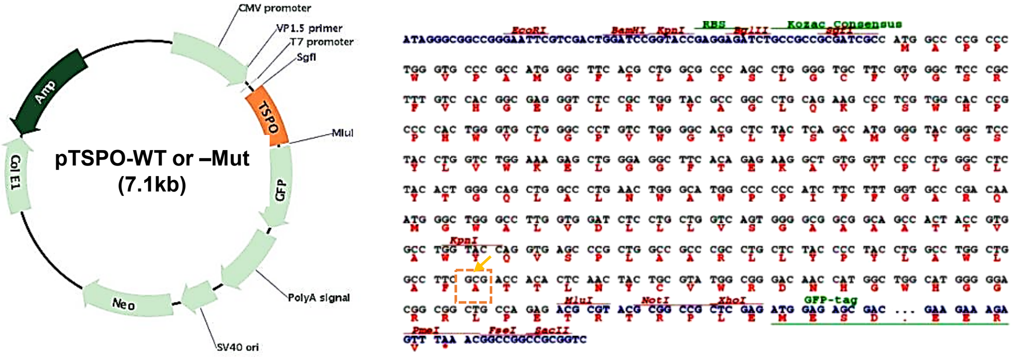

FIGURE S1. TSPO genotypes and classfication. (A) TSPO ligand binding classification. Single nucleotide polymorphism rs6971 TSPO (C→T point mutation; rs6971 A147T; Ala to Thr at amino acid 147 of TSPO) influences the binding affinity of TSPO ligands. TSPO with Thr/Thr at amino acid 147 shows a lower binding affinity to its ligand. TSPO ligand binding phenotypes are HAB or LAB, which indicate high- or low-affinity binding of ligands, respectively *Adapted from ref 32, 33. (B) Expression vector design for TSPO mutagenesis.
